# ADP as a novel stimulus for NLRP3-inflammasome activation in mice fails to translate to humans

**DOI:** 10.1007/s11302-023-09953-y

**Published:** 2023-07-06

**Authors:** Julius Wissemann, Adrian Heidenreich, Helene Zimmermann, Juliane Engelmann, Jasper Jansen, Dymphie Suchanek, Dirk Westermann, Dennis Wolf, Peter Stachon, Julian Merz

**Affiliations:** Cardiology and Angiology, Medical Center, University Heart Center Freiburg-Bad Krozingen, University of Freiburg, Freiburg, Germany

**Keywords:** Inflammation, NLRP3, Adenosine Diphosphate, Purinergic Receptor, PBMCs, P2Y1

## Abstract

The NLRP3-inflammasome is a cytosolic multiprotein complex that triggers an inflammatory response to certain danger signals. Recently adenosine diphosphate (ADP) was found to activate the NLRP3-inflammasome in murine macrophages via the P2Y_1_ receptor. Blockade of this signaling pathway reduced disease severity in a murine colitis-model. However, the role of the ADP/P2Y_1_-axis has not yet been studied in humans. This present study confirmed ADP-dependent NLRP3-inflammasome activation in murine macrophages, but found no evidence for a role of ADP in inflammasome activation in humans. We investigated the THP1 cell line as well as primary monocytes and further looked at macrophages. Although all cells express the three human ADP-receptors P2Y_1_, P2Y_12_ and P2Y_13_, independent of priming, neither increased ASC-speck formation could be detected with flow cytometry nor additional IL-1β release be found in the culture supernatant of ADP stimulated cells. We now show for the first time that the responsiveness of monocytes and macrophages to ADP as well as the regulation of its purinergic receptors is very much dependent on the species. Therefore the signaling pathway found to contribute to colitis in mice is likely not applicable to humans.

## Introduction

Inflammasomes are cytosolic multiprotein complexes that play a vital role in inflammation and the innate immune defense. The Nod-like receptor Family Pyrin Domain Containing 3 (NLRP3)-inflammasome is the most studied inflammasome and includes the pattern recognition receptor NLRP3, the adaptor protein “apoptosis associated speck-like protein containing a caspase activation and recruitment domain” (ASC) and the effector caspase-1 [1]. Aberrant regulation of inflammasome activation is implicated in various diseases [2–5].

While different cells have been proposed to be inflammasome competent, monocytes and macrophages express high levels of NLRP3 and are the major cell type showing inflammasome activation [6–9]. Prior to inflammasome assembly the inflammasome subunits are up-regulated via pro-inflammatory NF-κB-dependent pathways [10]. Inflammasome assembly can be triggered by a variety of stimuli, especially pathogen and damage associated molecular patterns (PAMPs/DAMPs), including extracellular nucleotides such as adenosine triphosphate (ATP). Downstream of their recognition receptor these agents elicit the activation of the NLRP3-inflammasome by promoting the polymerization of inflammasome subunits to form a multiprotein speck-like complex. This assembled inflammasome recruits and activates pro-caspase-1 by autocleavage to fully active caspase-1, leading to the processing of pro-IL-1β and pro-IL-18 into their mature cytokine forms. IL-1β and IL-18 are then released during a highly inflammatory form of cell death called pyroptosis, where pore-forming Gasdermin-D translocates into the plasma membrane [1].

Extracellular nucleotides such as ATP and ADP are important triggers for the assembly of the inflammasome after their release into the extracellular space upon cellular stress or death. Extracellular nucleotides orchestrate abundant immunological processes by activating a broad spectrum of nucleotide-specific purinergic receptors. Purinergic receptors are categorized into anti-inflammatory adenosine-specific P1 receptors and rather pro-inflammatory P2 receptors. P2 receptors can be further divided into seven ionotropic ATP-dependent P2X_1-7_ and eight G-protein-coupled, metabotropic P2Y receptors. In this context, ATP is a well-described activator of the NLRP3 inflammasome via activating the ionotropic purinergic receptor P2X_7_ [11–13]. Binding of ATP to P2X_7_ leads to K^+^-efflux which induces conformational changes in NLRP3, resulting in inflammasome assembly with subsequent release of pro-inflammatory cytokines [14]. Depletion of P2X_7_ blocks inflammasome activation in murine macrophages and ameliorates atherosclerosis by blocking lesional inflammasome activity in mice [15]. The P2Y receptors recognize different extracellular nucleotides with P2Y_1_, P2Y_12_ and P2Y_13_ being specific for ADP. Recent studies have shown that ADP also acts as a DAMP in a mouse model of ulcerative colitis and activates the NLRP3 inflammasome mainly through P2Y_1_ in murine bone marrow-derived macrophages (BMDMs). The other ADP receptors, P2Y_12_ and P2Y_13_, seem to play a lesser role in NLRP3 activation [16]. Regarding the human immune system, there is evidence of an ATP/P2X_7_ dependent inflammasome activation axis [17–19]. Nonetheless, the impact of a human ADP/P2Y_1_ dependent inflammasome activation axis remains unknown.

The aim of our work was to investigate whether the findings by Zhang et al*.* that implicated a role for ADP/P2Y_1_ in inflammasome activation are applicable to humans. If this turns out to be true, ADP and its receptors could be of therapeutic interest.

## Materials and Methods

### Murine bone marrow derived macrophages (BMDMs) cultivation and differentiation

ASC-citrine mice (RRID: ISMR_JAX:030744) carry a R26-CAG-ASC-citrine allele, which leads to constitutive expression of the ASC-citrine fusion protein from the Rosa26 locus. Citrine can be exited using lasers (Max. excitation: 515 nm; Max. emission 529 nm). Upon inflammasome activation, assembly of ASC into a speck leads to a bright and dense fluorescence signal [20].

Bone marrow was isolated and macrophages cultivated as previously described [21]. Briefly, bone marrow of the femurs was flushed through a 40-µm strainer with PBS and the cells cultivated in DMEM + 10% FBS + 1% P/S + 30 ng/ml murine M-CSF (PeproTech) for 6 days. On day 3, non-adherent cells were removed by a medium change.

### Murine PBMCs isolation and cultivation

1 ml of EDTA-blood was drawn through cardiac puncture and isolated using Lymphoprep™ following the manufacturers protocol (Stemcell). For stimulation, 5 × 10^5^ PBMCs were seeded in 400 µl RPMI 1640 medium (+ GlutaMAX™ + 10% FBS + 2% P/S) in a 48-well plate.

### THP1-ASC-GFP-cells cultivation and differentiation

THP-1-ASC-GFP (Invivogen) is a monocyte cell line carrying an ASC-GFP fusion construct, which is expressed under the control of an NF-κB inducible promotor [22]. Cells were handled according to manufacturer’s instructions and differentiated into macrophages according to a protocol by Park et al. [23]. Briefly, monocytes were cultured in growth medium with 5 ng/ml phorbol-12-myristat-13-acetat (PMA) (Invivogen) for 48 h and left to rest in test medium for 3 h prior to stimulation.

### Human PBMCs isolation, cultivation and differentiation in monocyte derived macrophages

10 ml of arterial EDTA-blood was collected from patients undergoing cardiac catheterization. Peripheral blood mononuclear cells (PBMCs) were isolated using SepMate™ and Lymphoprep™ following the protocol of the manufacturer (Stemcell). For stimulation, 5 × 10^5^ PBMCs were seeded in 400 µl RPMI 1640 medium (+ GlutaMAX™ + 10% FBS + 2% P/S) in a 48-well plate.

For differentiation to monocyte derived macrophages (MDMs), 1.5 × 10^6^ PBMCs were seeded into 24-well plates with 500 µl RPMI 1640 medium (+ GlutaMAX™) and incubated for 2.5 h to allow monocyte adherence. The medium was discarded along with the non-adherent cells and replaced with RPMI 1640 + GlutaMAX™ + 10%FBS + 2% P/S + 50 ng/ml human M-CSF (PeproTech). Cells were cultured for 7 days and half of the medium was renewed on day 3. On day 7, macrophages were differentiated by treatment with either 100 ng/ml lipopolysaccharide (LPS) from E.coli O55:B5 (Sigma) and 50 ng/ml interferone-γ (PeproTech) (M1), 20 ng/ml Interleukin-4 (PeproTech) (M2) or culture medium (M0) for 5 h.

### Stimulation of cells for inflammasome activation

For priming, cells were incubated with 100 ng/ml LPS for 4 h and the NLRP3-inflammasome was activated using 10 µM Nigericin (Invivogen), 5 mM ADP or ATP (Sigma) for 1 h. 3 µM of the NLRP3-specific inhibitor MCC950 (SellekChem) was added 30 min before LPS. 1 U/ml Apyrase from potatoes (Sigma) was added 1 h before ADP or LPS.

### Detection of inflammasome activation

Inflammasome activation was detected with flow cytometry using a method published by Sester et al. [24]. Upon inflammasome activation, cytosolic ASC aggregates into a dense speck. This leads to a decrease in width of the fluorescent signal, while its area remains unchanged.

### Flow cytometry

For ASC-speck detection, cells were scraped and stained in culture medium for 15 min at 37° C with anti-ASC (fluorophore: PE, human clone: HASC71, BioLegend; murine clone: D2W8U, Cell Signaling). For staining of surface-markers, cells were resuspended in FACS-Buffer (PBS-/- + 1% FBS + 0.5% BSA) and incubated with fluorophore-conjugated antibodies (BioLegend, if not stated otherwise) for 30 min at 4° C. Murine BMDMs were defined as CD45.2^+^ (PacBlue, 104), CD11b^+^ (APC-Cy7, M1/70), F4/80 + (PE-Cy7, BM8) and lineage^−^ (PE), which included CD3e (145-2C11, Invitrogen), CD19 (1D3, Invitrogen), NK-1.1 (PK136) and Ly6G (1A8). PMA-induced differentiation of THP1-ASC-GFP-cells was confirmed by the upregulation of CD11b (BV421, ICRF44, BD Biosciences) and CD14 (PE-Cy7, 63D3). Human MDMs were defined as CD45.2^+^ (PacBlue, 2D1), CD14^+^ (FITC, 63D3), CD11b^+^ (APC-Cy7, M1/70) and MERTK^high^ (PE, 590H11G1E3). Samples were analyzed using the BD FACSCanto™ II.

### Fluorescence microscopy

BMDMs were incubated with 1 µg/ml Hoechst33342 and Propidium Iodide (ImmunoChemistry) for 10 min to stain for viability, scraped and the suspension mounted onto microscope slides. Imaging was carried out using the Axio Imager Z2 and Zen 3.1 software (Zeiss).

### ELISA

Culture plates were centrifuged with 400 rcf for 5 min before collecting the supernatant. IL-1β levels were detected using the Human IL-1 beta/IL-F2 Quantikine ELISA Kit or the DuoSet ELISA (R&D systems) according to the manufacturer’s instructions. To keep data points within the linear range of the assay, samples were diluted up to 100-fold before assaying.

### Real-time PCR

RNeasy Mini Kit (Qiagen) was used according to the manufacturer’s instructions to isolate RNA. RNA concentration was measured with Nanodrop2000 (ThermoFisher) and 250 ng used for cDNA synthesis with the high-capacity cDNA reverse transcription kit (Applied Biosystems).

1 µl of cDNA was added to 10 µl 2X qPCR-BIO probe mix Lo-ROX, 7 µl nuclease free water, and 1 µl of the TaqMan assay (Thermofisher) for housekeeping (β-actin: Hs01060665_g1) as well as the respective target gene. Targets genes were P2Y_1_ (Hs00704965_s1), P2Y_12_ (Hs00224470_m1) and P2Y_13_ (Hs00256749_s1). The data was acquired in the C1000 Touch Thermocycler (Bio-Rad) and analyzed with the ddCT-method.

### WesternBlot

Cells were lysed in 100 µl RIPA-Buffer (ThermoFisher) with protease inhibitor (Roche). After addition of SDS- and β-mercaptoethanol containing loading buffer, standard western-blotting procedure was used. Proteins were separated using SDS-page and transferred to a nitrocellulose membrane. Primary antibodies were anti-P2Y_1_ (1:200, APR-009, alomone labs) and anti-β-actin (1:50.000, MAB8929, R&D Systems).

### Statistics

Using GraphPad Prism 8, data sets were tested for Gaussian distribution with the Shapiro–Wilk-test. In-vitro conditions were applied to each individual, therefore analysis of corresponding data sets was performed with a repeated measures one-way ANOVA followed by the Bonferroni post-hoc test to adjust for multiple comparisons.

## Results

### Validation of ADP-dependent NLRP3-activation in inflammasome reporter mice

**Fig. 1:**
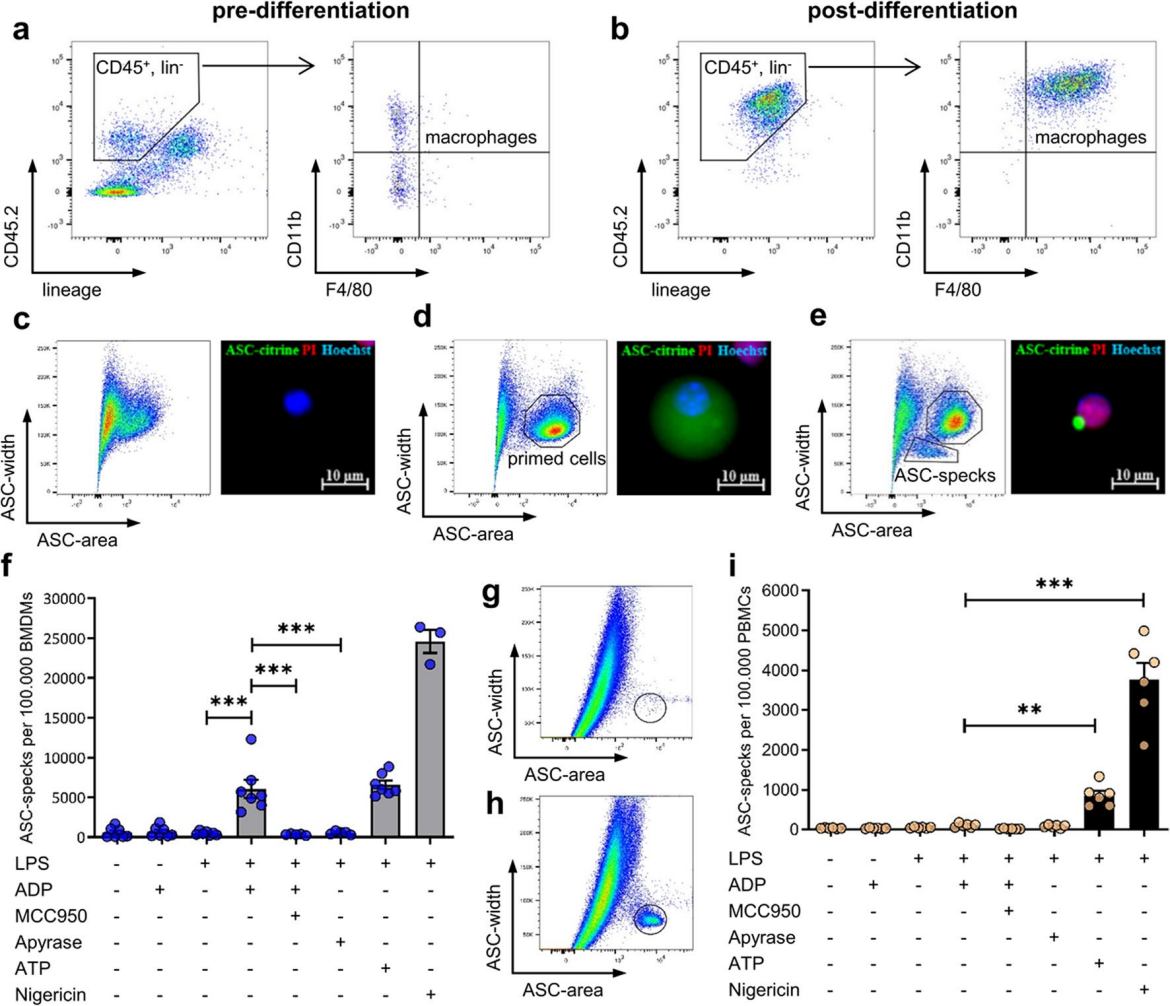
**Validation of ADP-dependent NLRP3 activation in inflammasome reporter mice**. Flow cytometric analysis of bone marrow isolated from C57BL6/J mice before (**a**) and after differentiation (**b**) to bone marrow derived macrophages (BMDMs) with 30 ng/ml macrophage colony-stimulating factor (M-CSF) for 6 days. Lineage staining contained antibodies recognizing CD3, CD19, NK1.1 and Ly6G. BMDMs from ASC-citrine mice were stimulated with 100 ng/ml lipopolysaccharide (LPS) for 4 h and 5 mM adenosine diphosphate (ADP), 5 mM adenosine triphosphate (ATP) or 10 µM Nigericin for 1 h. BMDMs with unprimed (**c**), primed (**d**) and assembled (**e**) inflammasomes were analyzed with fluorescence microscopy and flow cytometry. 1 µg/ml propidium iodide (PI) and Hoechst33342 (Hoechst) were added 10 min before imaging. ASC-specks were quantified with flow cytometry in three independent experiments, *n* = 3–7 (**f**). 3 µM MCC950 was added 30 min before LPS, 1 U/ml Apyrase was added 1 h before ADP. Flow cytometric detection of ASC-specks using an ASC-antibody in unstimulated (**g**) or LPS + Nigericin (**h**) treated PBMCs of C57BL6/J mice. ASC-specks were quantified with flow cytometry in two independent experiments, *n* = 6 (**i**). Statistical analysis was performed with repeated measures one-way ANOVA followed by Bonferroni corrected post hoc test. * *p* ≤ 0.05, ** *p* ≤ 0.01*,* *** *p* ≤ 0.001

BMDM differentiation after M-CSF stimulation was assessed using flow cytometric analysis. In the population of CD45^+^Lin^−^ leukocytes from freshly isolated murine bone marrow, no F4/80^+^ macrophages could be detected (Fig. 1a). Six days after cultivation with M-CSF the cell culture completely consisted of CD45^+^Lin^−^CD11b^+^F4/80^+^ BMDMs (Fig. 1b). Unstimulated macrophages from ASC-Citrine mice did not show a relevant ASC-signal in both flow cytometry and fluorescence microscopy (Fig. 1c). LPS primed macrophages revealed homogenously distributed ASC-citrine signals throughout the cytoplasm, resulting in an ASC-width^high^area^high^ distribution in flow cytometry (Fig. 1d). After stimulation with ADP, the ASC-citrine signal was relocated to the perinuclear zone, resulting in a dense ASC-speck with a strong fluorescent signal. Accordingly, flow cytometry detects ASC-specks as an ASC-width^low^area^high^ signal. ASC aggregation was accompanied by the nuclear co-localization of propidium iodide (Fig. 1e). Flow cytometric quantification revealed that neither LPS nor ADP alone had the potential to induce ASC-speck formation in murine BMDMs. Intriguingly, ADP stimulation after LPS priming led to an increase of ASC-specks. This ADP-dependent ASC-speck formation in primed BMDMs could be completely blocked by the specific NLRP3-inhibitor MCC950. Furthermore, ADP degradation by pre-treatment with apyrase also prevented ASC-speck formation in primed BMDMs. The effect size of ADP-induced inflammasome activation in BMDMs was comparable to that of ATP. Nigericin activates the NLRP3-inflammasome independent of receptors and led to the most abundant ASC-speck formation (Fig. 1f). In PBMCs from C57BL6/J mice, ASC-specks were detected using an ASC-antibody, only showing an ASC-width^low^area^high^ population upon inflammasome activation. (Fig. 1g, h) Primed cells are not visible, as intact cells are impermeable to the antibody. Quantification revealed, different to ATP and Nigericin no relevant increase in ASC-specks upon ADP stimulation of LPS-primed PBMCs.

### ADP does not induce ASC-specks in THP1 ASC-GFP-cells and THP1 derived macrophages

**Fig. 2:**
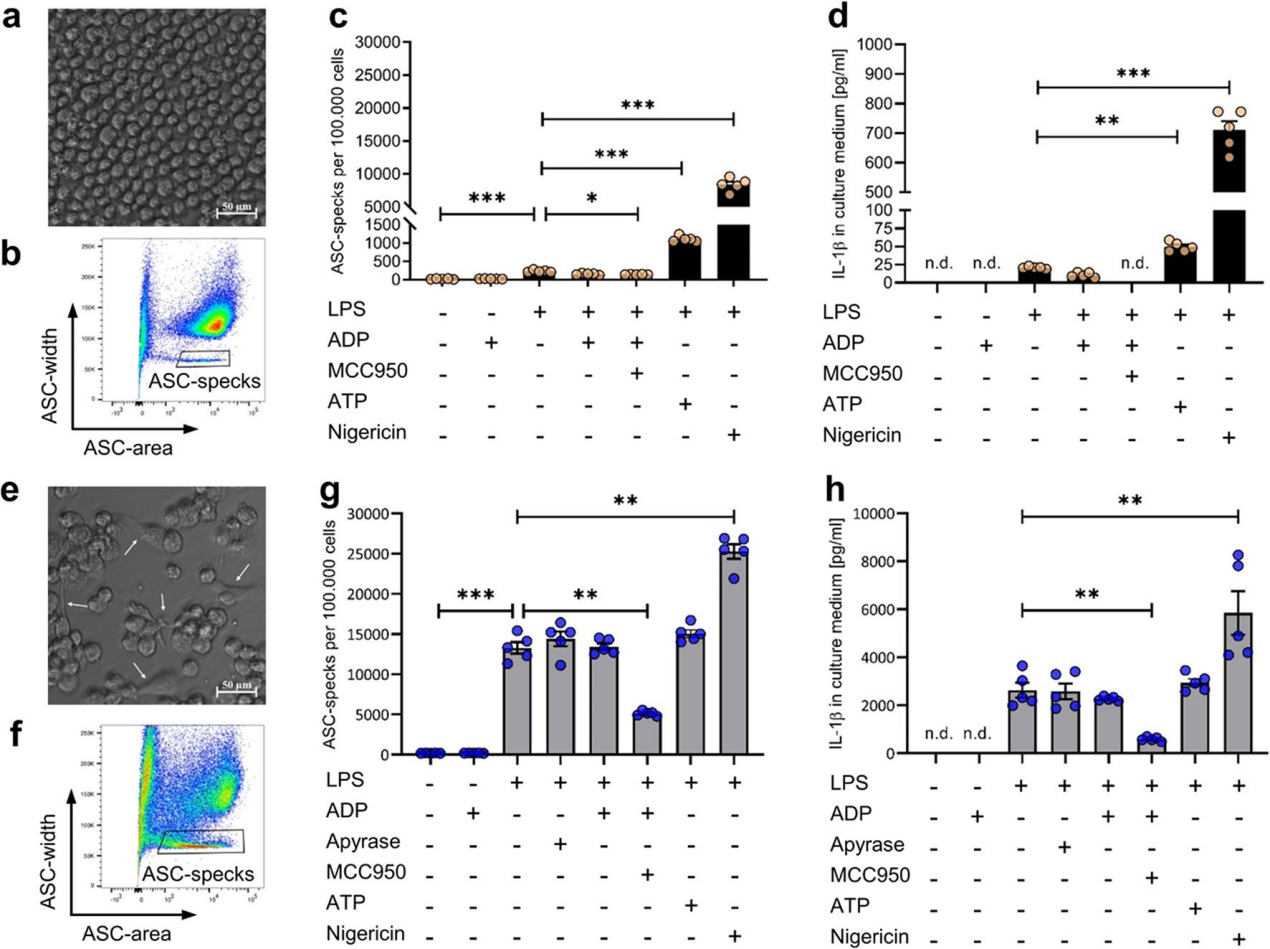
**ADP does not induce ASC-specks in THP1-ASC-GFP cells.** THP1-ASC-GFP cells before (**a**) and after differentiation to macrophages (**e**) with 5 ng/ml phorbol-12-myristat-13-acetat (PMA) for 48 h. White arrows point at morphological changes. Flow cytometric ASC-speck detection after stimulation with LPS (100 ng/ml for 4 h) before (**b**) and after differentiation (**f**). Quantification of ASC-specks detected with flow cytometry before (**c**) and after differentiation (**f**), one experiment with *n* = 5 each. Quantification of IL-1β release detected with ELISA before (**d**) and after differentiation (**h**) in these cells. ADP/ATP (5 mM) or Nigericin (10 µM) were added after LPS for 1 h. 3 µM MCC950 was added 1 h before LPS. Apyrase was added 1 h before LPS. Statistical analysis was performed with repeated measures one-way ANOVA followed by Bonferroni corrected post hoc test. * *p* ≤ 0.05, ** *p* ≤ 0.01, *** *p* ≤ 0.001

To investigate the ADP-dependent inflammasome activation capacity on human cells, further experiments were performed in a monocyte-like inflammasome reporter cell line called THP1 ASC-GFP (Fig. 2a). Flow cytometric detection of THP-1 ASC-GFP derived ASC-specks could again be detected as an ASC-width^low^area^high^ population (Fig. 2b). Sequential stimulation of THP1 ASC-GFP cells with LPS followed by either ATP or nigericin stimulation resulted in a strong ASC-speck formation (Fig. 2c) and subsequent IL-1β release (Fig. 2d). The LPS-nigericin sequence promoted a nearly tenfold increased ASC-speck formation compared to the LPS-ATP sequence. In contrast, sequential stimulation of THP1 ASC-GFP cells with LPS followed by ADP stimulation did not promote the formation of ASC-specks and coherently did not induce IL-1β release. In order to investigate the ADP-dependent inflammasome activation potential in human macrophages, we next differentiated the THP1 cells with PMA to THP1 derived macrophages (Fig. 2e). In comparison to undifferentiated THP1 monocytes, the overall ASC-speck formation capacity was increased in PMA-differentiated THP1 macrophages (Fig. 2f). Stimulation with LPS alone already resulted in a strong ASC-speck formation and IL-1β production (Fig. 2h) which could be blocked partially by the specific NLRP3-inhibitor MCC950 (Fig. 2f). LPS-induced inflammasome activation was not reduced in the presence of apyrase. Nevertheless, neither additional ATP nor ADP stimulation resulted in increased inflammasome activation. Only stimulation with Nigericin resulted in an increase of the ASC-speck formation and IL-1β release.

### ADP does not activate the NLRP3-inflammasome in human peripheral blood mononuclear cells (PBMCs)

**Fig. 3:**
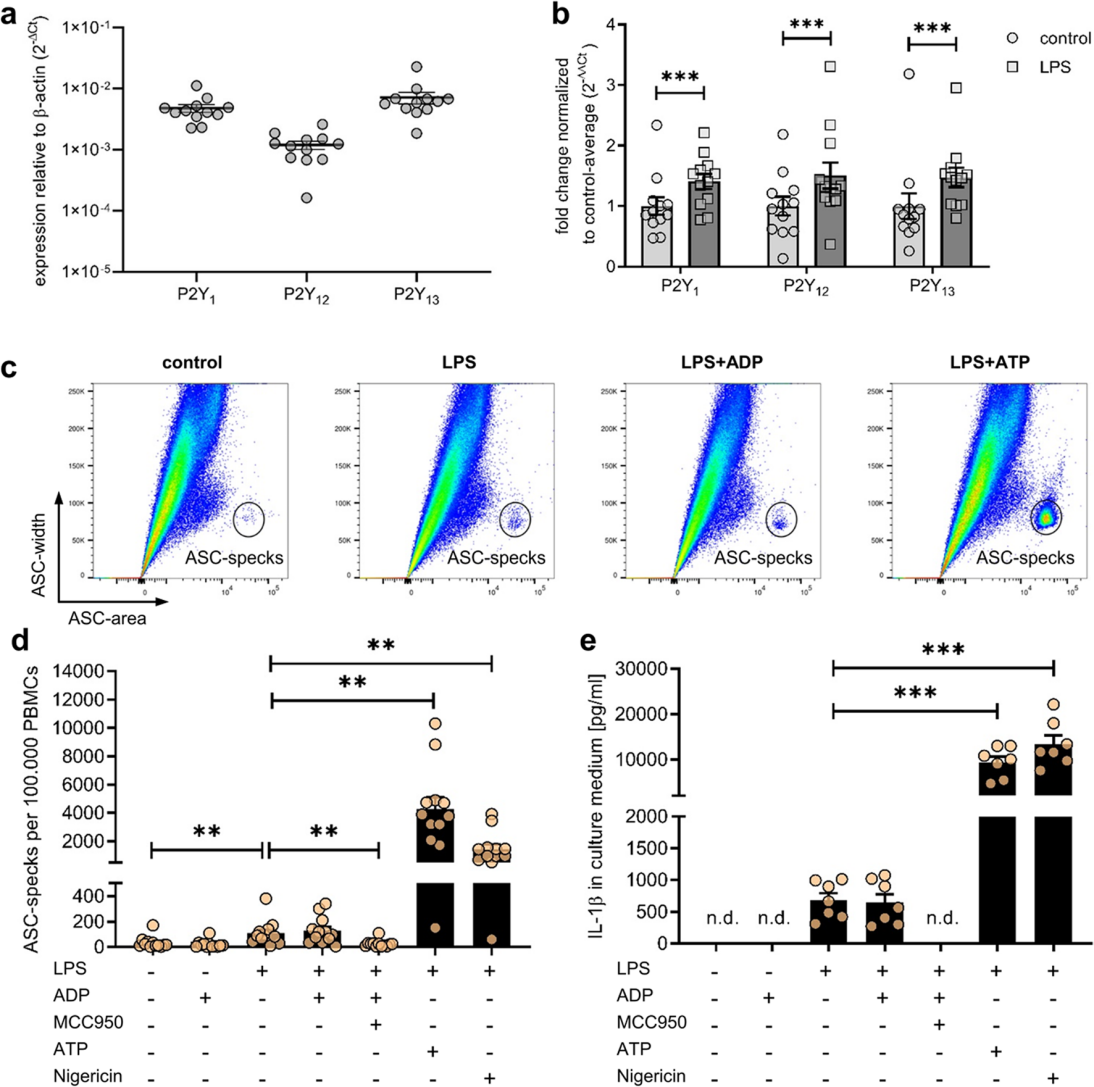
**ADP does not activate the NLRP3-inflammasome in human peripheral blood mononuclear cells (PBMCs).** Expression of ADP-receptors in untreated PBMCs as determined by quantitative PCR, four independent experiments, *n* = 12 (**a**). Regulation of receptors in these PBMCs after treatment with 100 ng/ml LPS for 4 h (**b**). Flow cytometric analysis of ASC-specks in PBMCs after stimulation with LPS for 4 h and 5 mM ADP, 5 mM ATP or 10 µM Nigericin for 1 h (**c**). Quantification of ASC-specks detected with flow cytometry in four independent experiments, *n* = 12 (**d**). 3 µM MCC950 was added 30 min before LPS. ELISA of cell culture supernatant from three independent experiuments for quantification of IL-1β release, *n* = 7(**e**). Statistical analysis was performed with repeated measures one-way or two-way ANOVA followed by Bonferroni corrected post hoc test. ** *p* ≤ 0.01, *** *p* ≤ 0.001

Next, we aimed to investigate the potential of ADP as an inflammasome activation stimulus in human primary cells. First, we examined the expression of all three ADP receptors P2Y_1_, P2Y_12_ and P2Y_13_ in human PBMCs (Fig. 3a). All of these ADP receptors were expressed and even upregulated following stimulation with LPS (Fig. 3b). Using a human anti-ASC antibody, the characteristic ASC width^low^area^high^ population again was detectable (Fig. 3c). Following LPS stimulation there was a detectable increase in the number of ASC-specks per 100.000 PBMCs compared to unstimulated PBMCs (Fig. 3d). This increase could be blocked by the addition of MCC950 and no further increase was detectable after LPS stimulation upon additional incubation with ADP. Nevertheless, additional incubation with ATP or Nigericin after LPS stimulation led to a more than 40-fold increased production of ASC-specks compared to LPS alone. Accordingly, the formation of ASC-specks in human PBMCs was accompanied by the secretion of IL-1β (Fig. 3e). Similar to the ASC-speck production, LPS alone led to a detectable increase of IL-1β. Again, only additional ATP or Nigericin stimulation but not ADP resulted in an increased production of IL-1β coherent with the aforementioned increased ASC-speck production. In human PBMCs MCC950 was able to block LPS-dependent IL-1β production completely.

### ADP-receptors are upregulated in anti-inflammatory human monocyte-derived macrophages (MDMs)

**Fig. 4:**
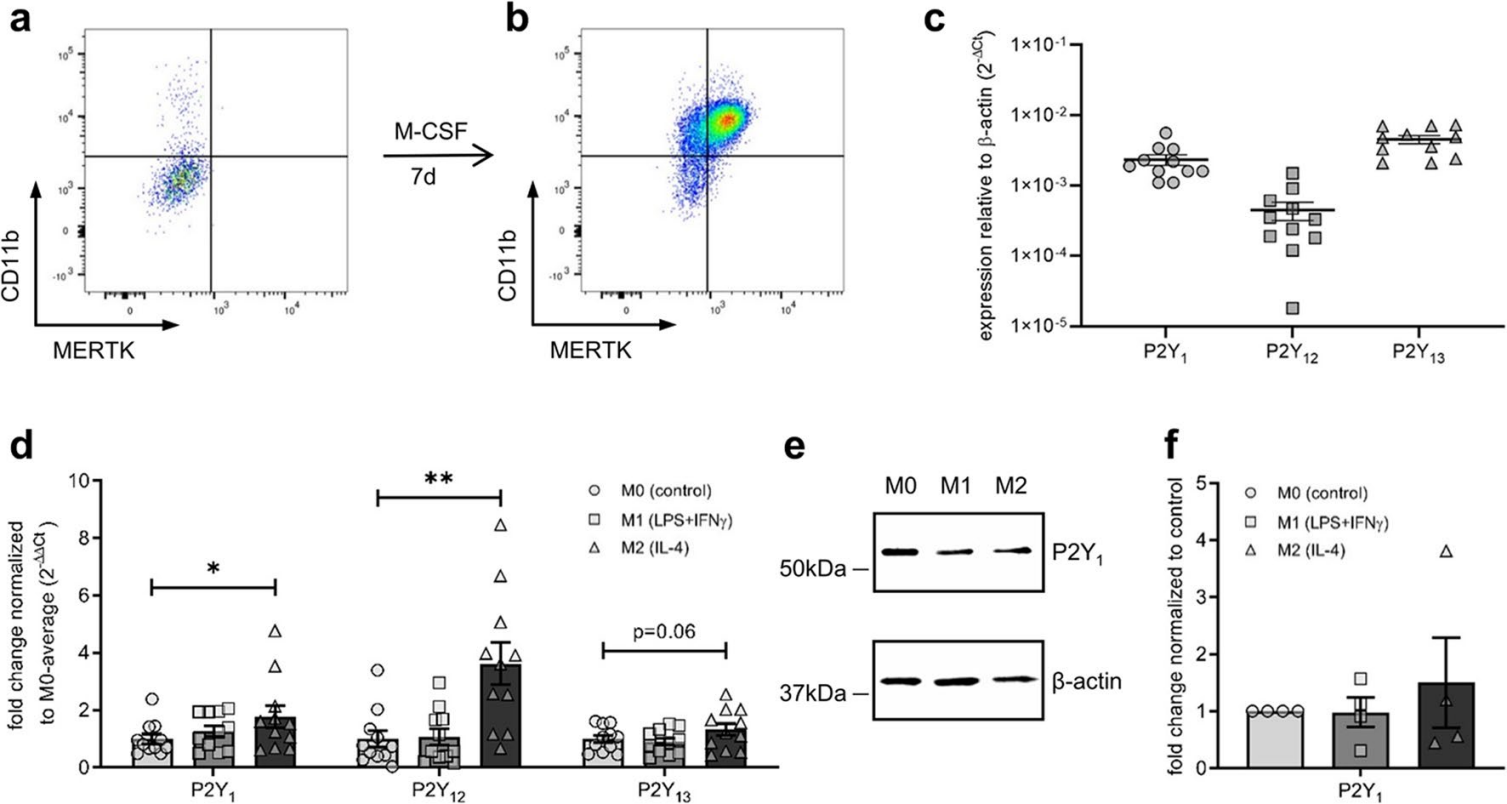
**ADP-receptors are upregulated in anti-inflammatory monocyte-derived macrophages (MDMs).** Expression of macrophage markers CD11b and MERTK on CD14.^+^ cells after isolation of human PBMCs (**a**) and after 7 days of culture with 50 ng/ml M-CSF (**b**). Expression of ADP-receptors in untreated MDMs as determined by quantitative PCR, four independent experiments, *n* = 11 (**c**). Change in gene expression after differentiation of these MDMs with either 100 ng/ml LPS and 50 ng/ml interferone-γ (M1), 20 ng/ml IL-4 (M2) or culture medium (M0) for 5 h (**d**). P2Y_1_ was detected with western blotting (**e**) and level of expression adjusted for β-actin, *n* = 4 (**f**). Statistical analysis was performed with repeated measures one-way ANOVA followed by Bonferroni corrected post hoc test. * *p* ≤ 0.05, ** *p* ≤ 0.01

Because undifferentiated PBMCs did not show any ADP-dependent ASC-speck formation, we next wanted to test this new potential inflammasome activation axis in human macrophages. Therefore, isolated PBMCs were stimulated with M-CSF for seven days. Flow cytometric analysis of CD14, CD11b and MerTK was used as cell surface markers of PBMC-derived macrophages. Native CD14^+^ PBMCs only show low cell surface levels of CD11b and MerTK (Fig. 4a), whereas M-CSF driven differentiation leads to an increase of both CD11b and MerTK (Fig. 4b). PBMC-derived macrophages express all three ADP receptors P2Y_1_, P2Y_12_ and P2Y_13_ (Fig. 4c). Macrophages can be further differentiated with LPS and IFN-γ into rather pro-inflammatory M1 and with IL-4 into rather anti-inflammatory M2 macrophages. In contrast to undifferentiated PBMCs there was no upregulation of the ADP-receptors P2Y_1_, P2Y_12_ and P2Y_13_ upon M1 differentiation with LPS and IFN-γ in PBMC-derived macrophages (Fig. 4d). Intriguingly, IL-4-driven M2 differentiation led to the up-regulation of all three ADP-receptors. Because recent studies postulated P2Y_1_ as the main purinergic receptor for ADP-dependent inflammasome activation [16], we next examined the protein levels of this receptor in human PBMC-derived macrophages. The purinergic ADP-receptor P2Y_1_ was detectable in undifferentiated, M1- and M2-differentiated PBMC-derived macrophages (Fig. 4e). The marginal transcriptional changes corresponded with a similar trend on protein level (Fig. 4f).

### ADP does not activate the NLRP3-inflammasome in human monocyte derived macrophages

**Fig. 5:**
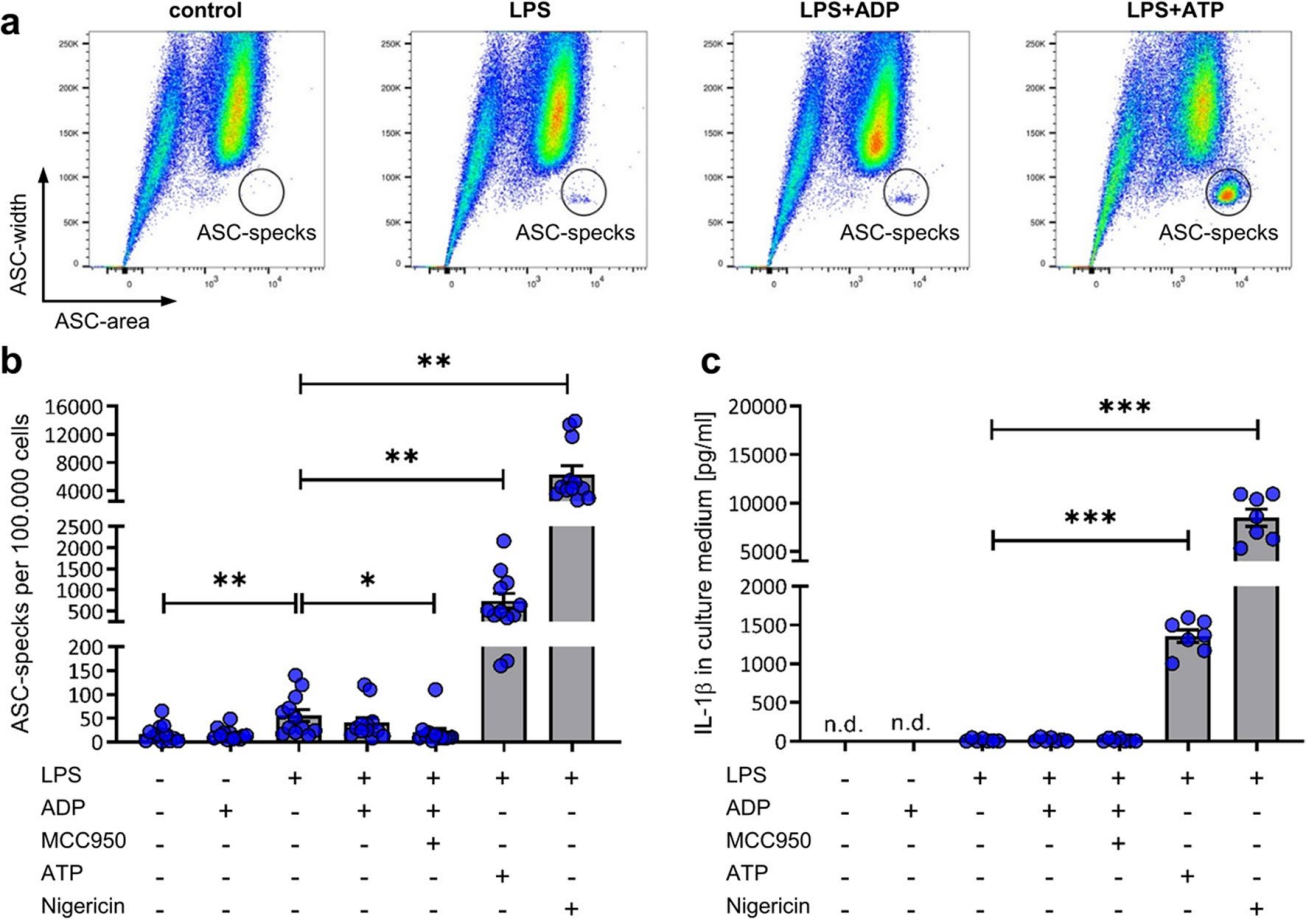
**ADP does not activate the NLRP3-inflammasome in human monocyte derived macrophages (MDMs).** Flow cytometric analysis of ASC-specks in MDMs after stimulation with 100 ng/ml LPS for 4 h and 5 mM, 5 mM ATP or 10 µM Nigericin for 1 h (**a**). Quantification of ASC-specks detected in four independent experiments, *n* = 12 with flow cytometry (**b**). 3 µM MCC950 was added 30 min before LPS. ELISA of cell culture supernatant from three independent experiments, *n* = 7 for quantification of IL-1β release (**c**). Statistical analysis was performed with repeated measures one-way ANOVA followed by Bonferroni corrected post hoc test. * *p* ≤ 0.05, ** *p* ≤ 0.01, *** *p* ≤ 0.001

Because the postulated main driver of ADP-dependent inflammasome activation P2Y_1_ was detectable in PBMC-derived macrophages both on the expressional and protein level, we finally challenged PBMC-derived macrophages to activate the inflammasome upon ADP stimulation. As already seen in PBMCs, the characteristic ASC width^low^area^high^ population was again detectable in PBMC-derived macrophages (Fig. 5a). Stimulation with LPS alone led to a very small increase of the ASC-speck population, which could be blocked with the NLRP3-specific inhibitor MCC950 (Fig. 5b). Nevertheless, ADP stimulation did not result in an increased ASC-speck formation in LPS-primed PBMC-derived macrophages. Only LPS priming followed by ATP or Nigericin stimulation resulted in a strong ASC-speck production. In unstimulated or ADP-treated PBMC-derived macrophages no IL-1β was detectable (Fig. 5c). Upon LPS stimulation either with or without ADP stimulation only minimal concentrations of IL-1β were measurable in the supernatant of PBMC-derived macrophages with MCC950 failing to block this effect. Stimulation of LPS-primed PBMC-derived macrophages with ATP or Nigericin led to a strong IL-1β signal consistent with abundant ASC specks.

## Discussion

Purinergic signaling in NLRP3-inflammasome activation plays an important role in a variety of inflammatory diseases such as atherosclerosis [15, 25], diabetes [26] or other inflammatory disorders [27]. Inflammasome activation research mainly focused on the ATP/P2X_7_-axis, unveiling its relevance in both rodents and humans [28]. Zhang et al*.* were the first to postulate that the ADP/P2Y_1_-axis activates the NLRP3-inflammasome in mice and implicated its relevance in experimental colitis. Following that discovery, they suggested ADP/P2Y_1_ as a potential drug target for inflammatory bowel disease [16]. However, regarding the NLRP3-inflammasome, significant mechanistic differences between species such as the dispensability of priming in human but not in murine monocytes have already been shown in the past [29]. Therefore, this study aimed to investigate the translatability of this novel described ADP/P2Y_1_-axis to the human system.

In order to quantify inflammasome activation, we used a novel flow cytometric based gating strategy to detect ASC-speck formation [24]. Using this new method, we were able to reproduce the finding of an ADP-dependent inflammasome activation axis in LPS-primed murine BMDMs [16]. Additionally, our study showed a clear NLRP3-dependency of this ADP driven pathway by successfully blocking ASC-speck formation with the selective NLRP3 inhibitor MCC950 [30, 31]. This confirmed the results from Zhang et al. and further strengthened the hypothesis of ADP as another NLRP3-inflammasome activation stimulus besides the well-characterized ATP-axis in the murine system. We further investigated ASC-speck formation in murine PBMCs. In this context, ADP failed to form ASC-specks after LPS stimulation. Intriguingly, subsequent ATP and Nigericin administration to LPS-primed murine PBMCs produced a detectable ASC-speck formation. This showed that ADP acts as inflammasome activation stimulus in murine macrophages but not in murine PBMCs.

Previous studies have shown that sole LPS stimulation promotes IL-1β release from human PBMCs but not from PBMC-derived macrophages [32–34]. In the present study, we were able to reproduce these findings in primary human monocytes and macrophages. Furthermore, we showed for the first time that LPS-dependent IL-1β release in human PBMCs is completely NLRP3 dependent as this effect is abolished by MCC950. Intriguingly, both human PBMCs and MDMs showed ASC-speck formation with LPS alone, which was blocked by MCC950. In contrast to PBMCs however, the LPS-induced ASC-speck formation in MDMs was not accompanied by significant release of mature IL-1β. Due to this, we hypothesize that both PBMCs and MDMs were able to activate the NLRP3 inflammasome under LPS-only conditions but only PBMCs respond with relevant IL-1β release. One explanation could be the rather anti-inflammatory differentiation stimulus of M-CSF with minor expression drive and release of IL-1β upon LPS stimulation [35, 36]. Interestingly, the responsiveness of PMA differentiated THP1-macrophages to LPS was so profound that only subsequent addition of Nigericin but not ATP was able to induce more ASC-specks and IL-1β release. This increased responsiveness of THP1 cells to LPS after PMA treatment, even when followed by a 24 h resting period, had previously been shown [37]. The presence of apyrase during incubation with LPS did not reduce inflammasome activation, indicating that the mechanism by which LPS activates the inflammasome is independent of extracellular ATP and ADP. Further studies are necessary to unravel the molecular mechanisms of LPS induced inflammasome activation and the differing response from monocytes and macrophages.

Both ATP and nigericin are routinely studied inflammasome activation stimuli in THP1 cells [38, 39], PBMCs [29] and PBMC-derived macrophages [40]. As expected, we found both an ATP and Nigericin-dependent increase of ASC-specks in all of those cell types after priming with LPS. Coherently, increased ASC-speck formation went hand in hand with mature IL-1β release from human monocytes and macrophages.

A recent study postulated that priming is dispensable for inflammasome activation in human PBMCs [29]. In this study however, neither PBMCs nor MDMs showed any inflammasome activation upon sole ADP stimulation. Although sequential ATP or Nigericin stimulation after LPS priming resulted in a strong ASC-speck formation with subsequent IL-β release, there was no detectable inflammasome activation after ADP stimulation of LPS-primed THP1 cells, PBMCs or MDMs. We postulate that in contrast to mice, neither human monocytes nor monocyte-derived macrophages can activate the NLRP3 inflammasome through ADP stimulation. A possible explanation could be species-dependent differences in the purinergic receptor repertoire and downstream intracellular pathways.

It has already been reported that mRNA of the ADP-receptors P2Y_1_, P2Y_12_ and P2Y_13_ is expressed in human monocytes and their derived cells [41–44]. While functional expression of P2Y_12_ and P2Y_13_ protein has been validated, there is only scarce evidence for P2Y_1_ protein levels in human monocytes or MDMs [45–47]. In this study, we were able to detect P2Y_1_ via immunoblotting in PBMC-derived macrophages. Previous studies showed that murine BMDMs only express P2Y_1_ and P2Y_13_ but not P2Y_12_ [21]. Polarization of murine BMDMs to a pro-inflammatory M1 or anti-inflammatory M2 phenotype revealed that P2Y_1_ expression is upregulated in M2 and absent in M1. Contrary, P2Y_13_ expression is mainly upregulated in M1 and downregulated in M2 macrophages [21]. Our current work provides evidence that the three human ADP-receptors are all upregulated in human PBMC-derived M-CSF derived macrophages after anti-inflammatory polarization, which has previously only been described for P2Y_12_ [48, 49]. These diverging findings regarding ADP receptor expression and regulation between mice and humans suggest that the response to ADP could differ in more than just inflammasome activation.

For mice, Zhang et al. proposed that activation of P2Y_1_ induces Ca^2+^ mobilization leading to phosphorylation of Extracellular Signal-regulated Kinase 5 (ERK5). This would mediate activation of Protein Tyrosine Kinase 2 (PYK2), which is then responsible for the phosphorylation of ASC that ultimately allows NLRP3 activation [16]. A similar role of PYK2 for NLRP3-inflammasome activation in human macrophages [50] as well as the involvement of ERK5 in M-CSF induced macrophage proliferation [51] have been shown. Whether the human P2Y_1_-receptor signaling pathway also induces the ERK5/PYK2 axis is currently unknown and needs further investigations. Furthermore, there could be a multitude of other promoting or inhibiting pathways involved in inflammasome regulation that differ between species [52]. For example, ectonucleotidases shape the strength of signaling pathways executed by extracellular nucleotides [53]. However, as ATP induces abundant ASC-specks, the influence of ectonucleotidases would not explain the absence of ADP-induced inflammasome activation. Future studies should aim to unravel the underlying mechanisms for differences in ADP-receptor signaling between different cell types as well as mice and humans.

The postulated mechanism of P2Y_1_-dependent inflammasome activation was investigated in a murine model of colitis. Especially for chronic inflammatory bowel disease, further immunomodulatory mechanisms have to be uncovered to find new therapeutic targets. Unfortunately, the postulated ADP-dependent inflammasome activation axis seems to be negligible in the human system. Yet, our study is limited by its in-vitro setting and missing heterogeneity of macrophages. While macrophages plasticity allows for a plethora of different functions in-vivo [54], ex-vivo analyses are often limited by only blood derived macrophages being available. Still, future studies could use different treatment conditions for macrophage differentiation to investigate a broader subset or even look at human bone marrow derived macrophages,

To conclude, ADP is unable to activate the inflammasome and does not induce ASC-speck formation in human monocytes and macrophages. Therefore, responsiveness to ADP and the regulation of its purinergic receptors are highly species-dependent. This highlights the need for caution when translating results obtained in the murine system to humans.

## Data Availability

The datasets generated and/or analysed during the current study are available from the corresponding author on reasonable request.
